# Chlorpyrifos Induces the Expression of the Epstein-Barr Virus Lytic Cycle Activator BZLF-1 via Reactive Oxygen Species

**DOI:** 10.1155/2015/309125

**Published:** 2015-07-14

**Authors:** Ling Zhao, Fei Xie, Ting-ting Wang, Meng-yu Liu, Jia-la Li, Lei Shang, Zi-xuan Deng, Peng-xiang Zhao, Xue-mei Ma

**Affiliations:** ^1^Beijing Environmental and Virus Cancer Key Laboratory, Beijing University of Technology, Beijing 100124, China; ^2^College of Life Science and Bioengineering, Beijing University of Technology, Beijing 100124, China

## Abstract

Organophosphate pesticides (OPs) are among the most widely used synthetic chemicals for the control of a wide variety of pests, and reactive oxygen species (ROS) caused by OPs may be involved in the toxicity of various pesticides. Previous studies have demonstrated that a reactivation of latent Epstein-Barr virus (EBV) could be induced by oxidative stress. In this study, we investigated whether OPs could reactivate EBV through ROS accumulation. The Raji cells were treated with chlorpyrifos (CPF), one of the most commonly used OPs. Oxidative stress indicators and the expression of the EBV immediate-early gene BZLF-1 were determined after CPF treatment. Our results show that CPF induces oxidative stress as evidenced by decreased malondialdehyde (MDA) level, accompanied by an increase in ROS production, DNA damage, glutathione (GSH) level, and superoxide dismutase (SOD) and catalase (CAT) activity. Moreover, CPF treatment significantly enhances the expression of BZLF-1, and the increased BZLF-1 expression was ameliorated by N-acetylcysteine (NAC) incubation. These results suggest that OPs could contribute to the reactivation of the EBV lytic cycle through ROS induction, a process that may play an important role in the development of EBV-associated diseases.

## 1. Introduction

Pesticides are widely used in almost every nation in the world. The indiscriminate use of pesticides in agriculture and for public health purposes has caused serious environmental and health problems [1]. Organophosphate pesticides (OPs) are one of the most commonly used pesticides, and their toxicity to humans and other nontarget species has caused increasing concern. OPs cause several harmful effects, including genotoxicity [2], hepatic dysfunction [3], embryo toxicity, teratogenicity, neurotoxicity [4], and neurobehavioral changes. Recent studies indicate that toxic manifestations induced by OPs may be associated with the enhanced production of reactive oxygen species (ROS) [5, 6]. Cells are equipped with several antioxidant agents comprising many enzymes, such as superoxide dismutase (SOD) and catalase (CAT), and nonenzymes, such as glutathione (GSH) and tocopherol. The antioxidant defense system protects cells from attack by ROS. Oxidative stress arises when the ROS production overwhelms the intrinsic antioxidant defenses. Excessive ROS can cause indiscriminate damage to biological molecules, leading to lipid peroxidation and protein and nucleic damage.

Epstein-Barr virus (EBV) is a ubiquitous human herpes virus that infects more than 90% of the world's population. The primary EBV infection occurs asymptomatically in childhood and typically persists throughout the life of the host. EBV is the cause of infectious mononucleosis [7] and is associated with specific forms of cancer, including Hodgkin's lymphoma [8], Burkitt's lymphoma [9], and nasopharyngeal carcinoma [10], and with autoimmune diseases [11]. In most asymptomatic carriers, the virus is periodically replicated, and the infectious virus is known as the EBV lytic cycle, which has been found to be associated with an increasing number of diseases, such as rheumatoid arthritis [12, 13] and infectious mononucleosis [7]. Lytic replication has been observed at the site of tumor development in posttransplant lymphoproliferative disorder (PTLD) [14] and Burkitt's lymphoma [15]. The EBV lytic cycle is characterized by the expression of one of the two EBV immediate-early genes, BZLF-1 and BRLF-1 [16]. These genes function as transcriptional activators and initiate an ordered cascade of lytic gene expression that results in the release of the infectious virus. In 2010, Lassoued et al. reported that oxidative stress induced by 0.2 mM H_2_O_2_ or 0.1 mM FeSO_4_ contributes to the reactivation of the EBV lytic cycle [17].

The aim of this study was to test the hypothesis that OPs reactivate the EBV lytic cycle via ROS accumulation, and we selected chlorpyrifos (CPF), a widely used OP, for the investigation. As a lipophilic molecule, CPF easily passes through the cell membrane. Recent studies have shown that CPF generates oxidative stress in rat and cell models [18, 19]. The effect of CPF on the reactivation of EBV was assessed by the measurement of the levels of BZLF-1 gene expression in Raji cells, a Burkitt's lymphoma-derived cell line. Simultaneously, the production of ROS, the degree of DNA damage, and the activity of antioxidants, such as SOD, CAT, and GSH, were measured to confirm the occurrence of oxidative stress.

## 2. Materials and Methods

### 2.1. Cell Culture

Raji cells derived from a human Burkitt's lymphoma which contains Epstein-Barr virus were obtained from the Cell Bank of Peking Union Medical College (PUMC, Beijing, China) and cultured in RPMI-1640 medium supplemented with 10% fetal bovine serum (FBS, Gibco, NY, USA) and 100 U of penicillin-streptomycin. The cells were maintained at 37°C in a humidified atmosphere of 5% CO_2_ (Thermo, USA). The culture medium was replaced every three days, and the subcultures were maintained at a ratio of 1 : 4.

### 2.2. Drug Treatment

CPF was obtained from Sigma-Aldrich (MO, USA). The CPF dilutions used for cell treatment were made from a CPF stock solution with a concentration of 0.1 M. Because CPF is a lipophilic molecule, its activity may be compromised if it binds to serum proteins; thus, the cells were transferred to a 1% serum medium before CPF treatment. The Raji cells were incubated with different concentrations of CPF (0, 50, 100, 150, and 200 *μ*M) at 37°C for 4 h. N-Acetylcysteine (NAC) (50 *μ*M), a well-known antioxidant, was added to culture medium with or without CPF for control.

### 2.3. Cell Proliferation Assay

The proliferation rate of the Raji cells was assessed using a quantitative colorimetric assay with MTT. Briefly, 3 × 10^4^ cells were seeded in 96-well plates. For dose-response studies, cells were treated with varying concentrations (0–1000 *μ*M) of CPF for 4 h at 37°C. After the incubation period, 10 *μ*L of MTT dye was added, and the cells were incubated for a further 4 h at 37°C. Then 100 *μ*L of DMSO was added and mixed thoroughly with the cells. The absorbance was measured at 570 nm using a Bio-Rad Model 680 microplate reader (Bio-Rad, USA). The IC_50_ value was calculated from dose-response curve using GraphPad Prism software.

### 2.4. Cell Cycle Analysis

The Raji cells were seeded in six-well plates at a seeding density of 10^6^ cells/mL. The cells were treated with different concentrations of CPF and incubated at 37°C for 4 h. The cells were harvested by trypsinization, collected, fixed in ice-cold 70% ethanol, and rewashed with cold PBS. The pellets were resuspended in 1 mL of a propidium iodide (PI) solution (0.1% Triton X-100, 0.1 mM EDTA, and 50 *μ*g/mL PI in PBS) containing 50 *μ*g/mL RNase and then incubated in the dark at 37°C for 30 min. The stained cells were incubated at room temperature for 30 min. The DNA content was analyzed using a BD FACSAria flow cytometer (BD Biosciences, USA). The results presented are representative of the data obtained from at least three independent experiments performed in triplicate.

### 2.5. Measurement of the Intracellular ROS Level

A total of 1 × 10^6^ cells were seeded in a poly-D-lysine coated plate and incubated with CPF in the presence or absence of the antioxidant NAC. The intracellular ROS was detected through the incubation of the cells with the CM-H_2_DCFDA fluorescence probe (Invitrogen, USA) at 37°C for 30 min in the dark. After removing the loading buffer, the cells were incubated for an additional 30 min in prewarmed fresh medium, and the fluorescent images were acquired with an Olympus microscope. The ROS content was quantified by flow cytometry.

### 2.6. RT-PCR Analysis

For the induction of the lytic cycle, 2 × 10^6^ cells were stimulated with different concentrations of CPF for 4 h. The cells were washed three times with phosphate buffer saline (PBS) and then incubated in fresh culture medium for 48 h. The total RNA from the cells was extracted using the RNeasy Mini Kit (Qiagen, USA) according to the manufacturer's instructions. The RNA was quantified through optical density measurement at 260 and 280 nm, and all of the samples had an A260/A280 ratio greater than 1.8. Equal quantities of the total RNA were reverse-transcribed into cDNA using a ProtoScript M-MuLV first-strand cDNA synthesis kit (NEB, USA). Real-time PCR was performed using a Maxima SYBR Green qPCR Master Mix (Fermentas, EU). The PCR primer sequences were obtained from qPrimerDepot (http://primerdepot.nci.nih.gov/) and synthesized by Shanghai Sangon (Shanghai, China). The data were analyzed through the comparative threshold cycle (Ct) method using GAPDH as the reference gene.

### 2.7. Comet Assay

The DNA damage was detected through an alkaline comet assay as described by Singh et al. [20] with minor modifications. Approximately 2500 cells were embedded between a layer of 1% low-melting-point agarose and a layer of 1% normal-melting-point agarose. The slides were immersed in a lysis solution (2.5 M NaCl, 100 mM EDTA, 10 mM Tris, pH 10, 1% Triton-X, and 10% DMSO) overnight at 4°C. To allow the DNA to unwind, the slides were placed in an alkaline buffer (0.3 M NaOH and 1 mM EDTA, pH 12) for 30 min at 4°C. After electrophoresis in a chilled alkaline buffer, the slides were neutralized with Tris-HCl buffer (400 mM, pH 7.4) and stained with Gel-Red (Biotium, USA). The DNA damage was assessed by the tail inertia of 100 cells per sample using a fluorescence microscope equipped with an image system running the Delta Sistemi software.

### 2.8. MDA, GSH, SOD, and CAT Assays

The intracellular MDA production was determined using a thiobarbituric acid reactive species (TBARS) assay kit (Cayman, USA). A total of 1 × 10^7^ cells were homogenized in PBS, and the lysates were incubated with TBA color reagents at 50°C for 60 min. The MDA-TBA adducts were then measured colorimetrically at 540 nm. The total protein concentration was determined through the Bradford assay (Tiangen, China), and the results are expressed as [MDA] in nmol per mg of protein. GSH was determined according to the recycling system through the reaction of 5,5′-dithiobis-(2-nitrobenzoic acid) (DTNB) and GSH (Dojindo, Japan). The concentration of GSH in the sample solutions was determined using a calibration curve and is expressed as nmol of GSH per mg of protein. The SOD activity was measured using an assay kit (Dojindo, Japan) according to the manufacturer's protocol. The CAT activity was assayed according to the method developed by Góth and is expressed as mU per mg of protein based on the rate of decrease of the hydrogen peroxide [21].

### 2.9. Statistical Analysis

The presented data were obtained from at least three independent experiments and are reported as the means ± the standard error of the mean (SEM). The statistical analyses of the data were performed using one-way analysis of variance (ANOVA). A two-tailed probability level of *p* < 0.05 indicates statistical significance. The nonsignificant results were reported as “ns.”

## 3. Results

### 3.1. Effect of CPF Exposure on Cell Proliferation

**Figure 1:**
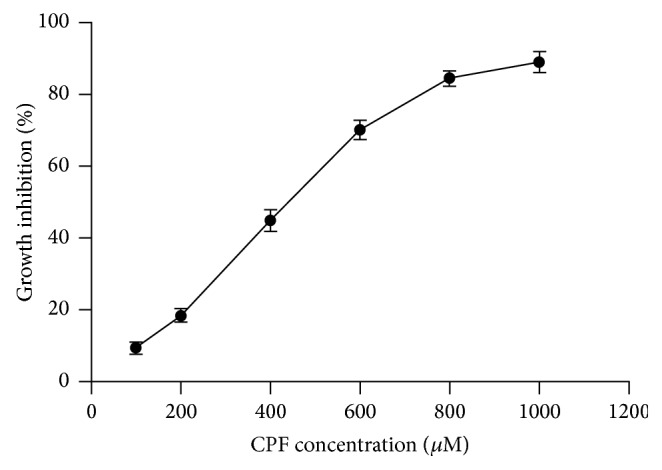
Effects of CPF treatment on Raji cell proliferation. The inhibitory rate of the Raji cell proliferation was determined after treatment with 0–1000 *μ*M CPF for 4 h.

The 4-h CPF treatment had no significant effects on the cell morphology. The proliferation rate of the Raji cells treated with 0–1000 *μ*M CPF for 4 h was determined using MTT assay. As shown in Figure 1, CPF could inhibit the proliferation of Raji cells in dose-dependent manner. The IC_50_ value for 4-h CPF treatment was 409.65 ± 25.65 *μ*M.

### 3.2. Effect of CPF Exposure on Cell Cycle Arrest

Cell cycle distribution was determined to elucidate the effect of CPF exposure on cell cycle arrest. The Raji cells were exposed to CPF (0–200 *μ*M) for 4 h and then evaluated through a PI-based cell cycle analysis. An increase in the CPF concentration resulted in a slight increase in the percentage of G0/G1 cells and a slight decrease in the percentage of cells in the G2/M phase. The changes in the percentages of cells in the G0/G1, G2/M, and S phases with different CPF concentrations showed no significant differences (Table 1).

### 3.3. CPF Treatment Induces ROS Production

**Figure 2:**
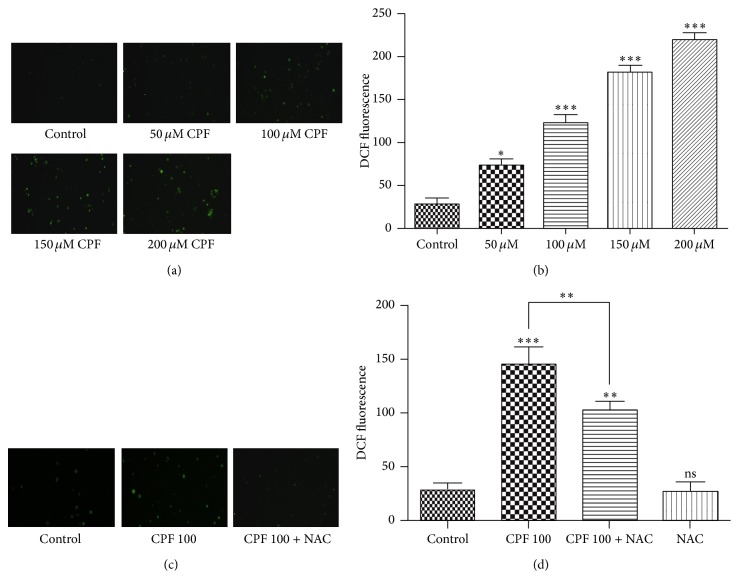
Generation of ROS by CPF. (a) Fluorescence microscopy imaging shows ROS production induced by CPF in different concentration. (b) Quantitative flow cytometry shows that ROS accumulation was induced in a concentration-dependent manner by CPF treatment. Effect of the antioxidant NAC (50 *μ*M) on the ROS accumulation induced by CPF (100 *μ*M) was evaluated by fluorescence microscopy imaging (c) and quantitative flow cytometry (d). The results are expressed as the means ± SEM. ^*∗*^ *p* < 0.05, ^*∗∗*^ *p* < 0.01, and ^*∗∗∗*^ *p* < 0.001.

In this study, we exposed Raji cells to CPF (0–200 *μ*M) for 4 h and measured the ROS levels using CM-H_2_DCFDA, a membrane-permeable nonfluorescent dye that can be oxidized by ROS to produce green fluorescence. The intracellular ROS content was observed by fluorescence microscopy imaging and then quantified by flow cytometry analysis. As indicated in Figure 2, ROS level was significantly increased (*p* < 0.05) compared with control after treatment with 50 *μ*M CPF for 4 h, and the ROS production was induced in a concentration-dependent manner by CPF treatment. The highest ROS level (7.89-fold versus control) was observed after treatment with 200 *μ*M CPF. To confirm these observations, the antioxidant agent NAC was used to block the ROS production, ROS level was significantly increased (*p* < 0.001) after treatment with 100 *μ*M CPF, and the NAC administration significantly inhibited the ROS accumulation (*p* < 0.01).

### 3.4. CPF Exposure Induces EBV Immediate-Early Gene BZLF-1 Expression

**Figure 3:**
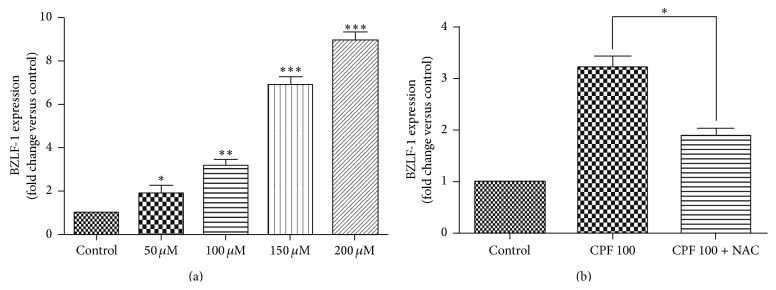
Induction of BZLF-1 gene expression by CPF in Raji cells. Raji cells were stimulated with CPF, and the BZLF-1 gene expression level was evaluated by real-time PCR. (a) CPF induces the EBV lytic cycle through the expression of BZLF-1 in a dose-dependent manner. (b) Effect of the antioxidant NAC (50 *μ*M) on the CPF- (100 *μ*M) induced increase in BZLF-1 expression. The results are expressed as the means ± SEM. ^*∗*^ *p* < 0.05, ^*∗∗*^ *p* < 0.01, and ^*∗∗∗*^ *p* < 0.001.

After determining the ROS production induced by the CPF treatments, the lytic cycle of the Raji cell line was analyzed based on the assessment of the expression of the EBV immediate-early gene BZLF-1 through RT-PCR. The results show that CPF exposure for 4 h can significantly induce the expression of BZLF-1 in a dose-dependent manner. In fact, the expression of BZLF-1 was increased 1.89-, 2.58-, 3.53-, and 9.25-fold at the concentration of 50, 100, 150, and 200 *μ*M CPF, respectively. To ascertain the involvement of the ROS in the CPF-induced EBV transcription, 50 *μ*M NAC was used as an antioxidant, and the results show that the expression of BZLF-1 was inhibited by NAC to some extent (Figure 3). These results suggest that CPF induces EBV reactivation through ROS accumulation.

### 3.5. CPF Treatment Induces DNA Damage

**Figure 4:**
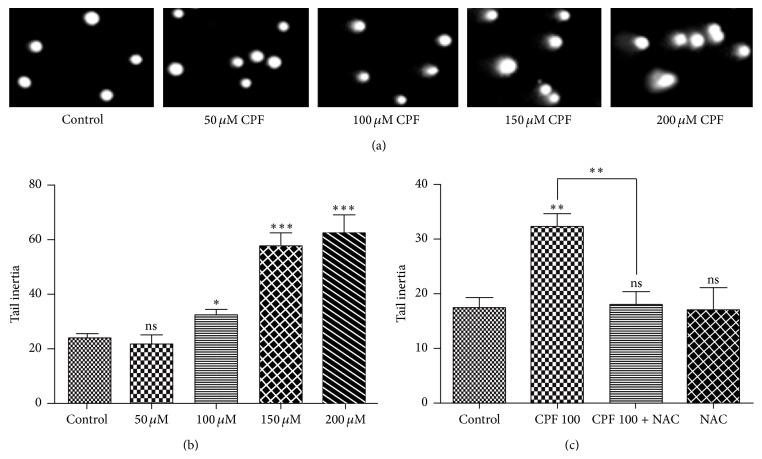
CPF-induced DNA damage in Raji cells. Raji cells were exposed to different concentrations of CPF. The comet assay was performed to assess the DNA oxidative damage. (a) Representative comet images for 0–200 *μ*M CPF treatment. (b) Exposure to CPF at any concentration with the exception of 50 *μ*M resulted in an obvious dose-dependent increase in the tail inertia. (c) The antioxidant NAC blocked the CPF-induced DNA damage. The results are expressed as the means ± SEM. ^*∗*^ *p* < 0.05, ^*∗∗*^ *p* < 0.01, and ^*∗∗∗*^ *p* < 0.001.

The DNA damage, which is represented by DNA single-strand breaks, was reflected by an increase in the tail moment. In this study, CPF treatment induced an obvious dose-dependent increase in the tail inertia, which is an important quantitative parameter for evaluating DNA damage (Figure 4). We found that CPF at a concentration of 50 *μ*M induces a slight, albeit not statistically significant, increase in the degree of DNA damage. However, CPF concentration ranging from 100 (*p* < 0.05) to 200 *μ*M (*p* < 0.001) resulted in significant DNA damage. In addition, we observed that the antioxidant NAC can block the DNA damage to a level that is 1.03-fold higher (without statistical significance) than the level observed in the control, whereas the DNA damage induced by CPF was 1.84-fold higher than the level observed in the untreated cells.

### 3.6. CPF Treatment Changes the Antioxidant System

**Figure 5:**
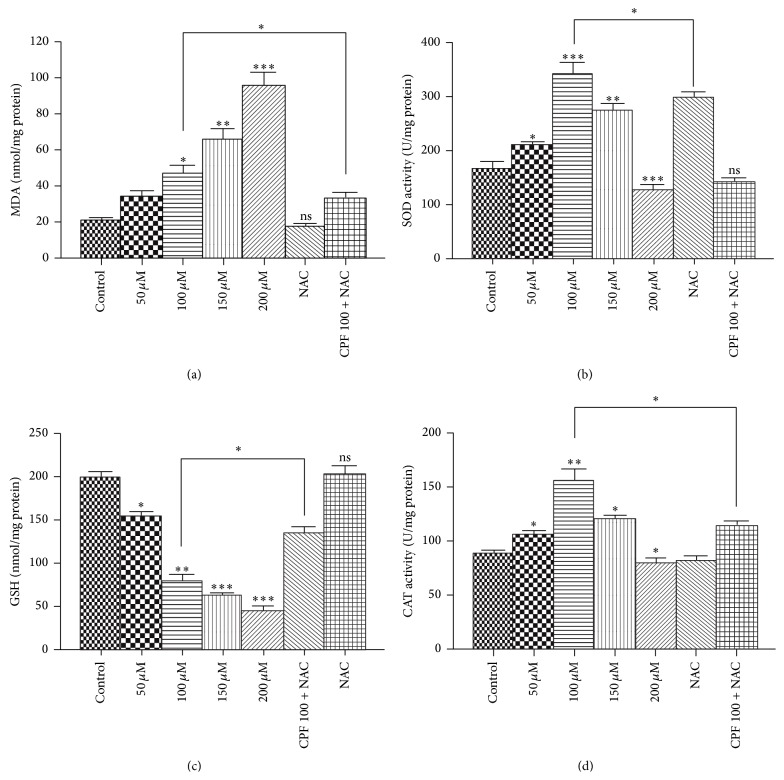
Oxidative stress was induced by CPF in Raji cells. Raji cells were exposed to different concentrations of CPF for 4 h. Oxidative stress markers, including the MDA and GSH levels and the activities of SOD and CAT, were determined to assess the CPF-induced oxidative stress. (a) MDA levels. (b) SOD activity. (c) GSH levels. (d) CAT activity. The results are expressed as the means ± SEM. ^*∗*^ *p* < 0.05, ^*∗∗*^ *p* < 0.01, and ^*∗∗∗*^ *p* < 0.001.

The oxidation of membrane lipids, which is one of the primary events in oxidative cellular damage, can be assessed by measuring MDA, a breakdown product of lipid peroxides. The incubation of Raji cells with CPF for 4 h caused a significant dose-dependent increase in the MDA levels. NAC pretreatment induced a significant decrease in the MDA level (33.72 ± 2.55 nmol/mg of protein) compared with the levels obtained with the CPF treatment (47.64 ± 3.43 nmol/mg of protein; Figure 5).

CAT and SOD play very important roles in protecting cells from oxidative damage. GSH is an important antioxidant because it prevents damage to biological macromolecules caused by the ROS. In this study, we observed that the GSH levels were significantly decreased by CPF treatment in a dose-dependent manner, and pretreatment with NAC effectively ameliorated this decrease. Exposure to CPF (50–150 *μ*M) resulted in a marked increase in the activity of SOD, whereas the activity of SOD was decreased after exposure to the high CPF concentration of 200 *μ*M. Similarly, the CAT activity was increased after CPF exposure (50–150 *μ*M), and the CPF concentration of 200 *μ*M caused a decrease in CAT activity. The NAC pretreatment effectively ameliorated the GSH reduction and the increases in the activities of SOD and CAT (Figure 5).

## 4. Discussion

The objective of this study was to determine whether CPF can reactivate the EBV lytic cycle in Raji cells and to identify the underlying mechanism. Our results show that the exposure of Raji cells to CPF leads to ROS production, DNA damage, lipid peroxidation, and antioxidant depletion. The expression of the EBV immediate-gene BZLF-1 was found to be triggered by CPF treatment, and NAC, a well-known antioxidant, can block EBV reactivation and alleviate oxidative stress in Raji cells. These findings indicate that CPF can induce an EBV lytic state via ROS accumulation.

Previous studies have demonstrated that latent EBV infection can be reactivated to lytic replication by treatment with various conditions, including hypoxia [22], radiation [23], drugs [24], and oxidative stress [25]. CPF, one of the most widely used OPs in agriculture, has been reported to cause oxidative stress. Because oxidative stress is implicated in EBV reactivation, we postulated that oxidative stress induced by CPF is involved in this process. Previous studies have demonstrated that cell cycle arrest is associated with EBV reactivation [26], and cell cycle arrest can be induced by 4-nonylphenol (NP) in a ROS-dependent manner [27]. We evaluated whether CPF can induce cell cycle arrest and thus contribute to EBV reactivation. Our results show that CPF treatment has no significant effect on cell cycle arrest. After a 4-h CPF treatment, EBV can be effectively converted into a lytic cycle, as indicated by the increased expression level of BZLF-1. These results indicate that cell cycle arrest is not involved in EBV reactivation, and CPF-induced ROS accumulation may play a direct role in this process.

EBV is a human herpesvirus that has been associated with some subgroups of non-Hodgkin's lymphomas (NHL), such as Burkitt lymphoma [28] and nasal NK/T-cell lymphoma [29]. An increase in the risk of developing NHL among individuals exposed to pesticides was reported [30–32]. In addition, a correlation of exposure to certain pesticides with increased titers of antibodies to EBV antigens was reported [33]. The results of the present study indicate the possibility that exposure to certain pesticides may induce EBV reactivation through ROS accumulation and thus contribute to EBV-related malignancies.

Oxidative stress is defined as an imbalance between ROS generation and antioxidant defense. To elucidate the oxidative stress induced by OPs, we detected the alteration in the levels of intracellular ROS and the antioxidant defense system. Our results demonstrate that the induction of intracellular ROS was elevated, as reflected by the increased fluorescence intensity detected by the CM-H_2_CDFDA probe. The quantitative assay performed in this study showed that the intracellular ROS content was increased 2.66-, 4.41- 6.50-, and 7.89-fold after treatment with the different concentrations of CPF. When ROS flood the antioxidant system, the excessive ROS trigger lipid peroxidation and nucleic acid damage. The levels of MDA, an indicator of lipid peroxidation, were increased in the CPF-treated cells compared with the controls. DNA damage has been identified to be a useful index of oxidative stress and a possible indicator of cancer [34]. ROS attack DNA bases or deoxyribose residues to produce damaged bases or strand breaks. The effect of CPF on DNA damage was measured through the comet assay, which is a very sensitive technique for the detection of DNA damage. The results of this study showed that all of the tested concentrations of CPF, with the exception of 50 *μ*M, induced a significantly high level of DNA damage.

The antioxidant system plays a key role in protecting cells from oxidative damage. ROS are chemically reactive molecules that predominantly include the superoxide anion (O^2−^), hydrogen peroxide (H_2_O_2_), and hydroxyl radicals. There are several enzymatic and nonenzymatic antioxidants in cells that are important for the scavenging of excessive toxic free radicals. SOD and CAT are the most important defense mechanisms against the toxic effects of ROS. In this study, the SOD and CAT activities were found to be increased significantly after CPF treatment compared with the levels found in the controls. The elevated SOD and CAT activities in the CPF-treated cells may serve as protective responses for the elimination of ROS. GSH maintains enzymes and other cellular components in a reduced state, and its content is a function of the balance between use and synthesis. The GSH level was found to be dose-dependently decreased in the CPF-treated cells, which suggests that GSH may be rapidly metabolized or oxidized in CPF-induced oxidative stress conditions.

To elucidate the involvement of the ROS in the CPF-induced EBV lytic cycle, we used the antioxidant agent NAC to block the ROS. Our results show that the incubation of Raji cells with NAC before CPF treatment can inhibit EBV reactivation. The oxidative stress was ameliorated by NAC, as evidenced by the observed decreases in the ROS content, SOD activity, DNA damage, MDA production, and GSH depletion. These results indicate that OPs are able to activate the EBV lytic cycle through ROS accumulation.

## 5. Conclusion

Chlorpyrifos treatment induces oxidative stress and significantly increases the expression of BZLF-1 in the Raji cell line. The antioxidant NAC mitigates the oxidative stress and elevated BZLF-1 expression induced by CPF. This evidence indicates that CPF treatment may reactivate the EBV lytic cycle via ROS accumulation. Thus, OPs may lead to an increase in the number of EBV-infected cells and favor the development of EBV-associated diseases.

## Figures and Tables

**Table 1:** Changes in cell cycle distribution after treatment with varying concentrations of CPF. The data (% of control) are shown as the means ± SD (*n* = 3).

CPF (*μ*M)	G1/G0 (%)	S (%)	G2/M (%)
0	39.08 ± 1.29	35.29 ± 0.93	25.63 ± 1.73
50	39.30 ± 1.39	36.46 ± 0.83	24.25 ± 1.20
100	39.77 ± 1.21	36.98 ± 1.04	23.25 ± 0.60
150	39.85 ± 0.67	37.36 ± 1.20	22.79 ± 1.32
200	41.73 ± 0.80	36.95 ± 0.98	21.32 ± 1.25
